# Sinking CO_2_ in Supercritical Reservoirs

**DOI:** 10.1029/2020GL090456

**Published:** 2020-11-29

**Authors:** Francesco Parisio, Victor Vilarrasa

**Affiliations:** ^1^ Chair of Soil Mechanics and Foundation Engineering, Institute of Geotechnics Technische Universität Bergakademie Freiberg Freiberg Germany; ^2^ Institute of Environmental Assessment and Water Research (IDAEA) Spanish National Research Council (CSIC) Barcelona Spain; ^3^ Mediterranean Institute for Advanced Studies (IMEDEA) Spanish National Research Council (CSIC) Esporles Spain; ^4^ Associated Unit: Hydrogeology Group UPC‐CSIC Barcelona Spain

**Keywords:** geologic carbon storage, supercritical geothermal systems, CO_2_ leakage, buoyancy, CO_2_ emissions reduction

## Abstract

Geologic carbon storage is required for achieving negative CO_2_ emissions to deal with the climate crisis. The classical concept of CO_2_ storage consists in injecting CO_2_ in geological formations at depths greater than 800 m, where CO_2_ becomes a dense fluid, minimizing storage volume. Yet CO_2_ has a density lower than the resident brine and tends to float, challenging the widespread deployment of geologic carbon storage. Here, we propose for the first time to store CO_2_ in supercritical reservoirs to reduce the buoyancy‐driven leakage risk. Supercritical reservoirs are found at drilling‐reachable depth in volcanic areas, where high pressure (*p* > 21.8 MPa) and temperature (*T* > 374°C) imply CO_2_ is denser than water. We estimate that a CO_2_ storage capacity in the range of 50–500 Mt yr^−1^ could be achieved for every 100 injection wells. Carbon storage in supercritical reservoirs is an appealing alternative to the traditional approach.

## Key Points

- We propose a novel geologic carbon storage concept that reduces the buoyancy‐driven CO_2_ leakage risk
- By injecting CO_2_ in reservoirs where the resident water stays in supercritical conditions, CO_2_ sinks because it is denser than pore water
- Supercritical reservoirs are found at relatively shallow depths between 3 and 5 km in deep volcanic areas

## 1 Introduction

Carbon Capture and Storage (CCS) is envisioned as a key technology to accomplish net negative carbon dioxide (CO_2_) emissions during the second half of the century and meet the COP21 Paris Agreement targets on climate change (Bui et al., 2018; Intergovernmental Panel on Climate Change [IPCC], 2018). However, CCS should overcome two main hurdles, namely, the risks of induced seismicity (Vilarrasa & Carrera, 2015; Zoback & Gorelick, 2012) and CO_2_ leakage (Lewicki et al., 2007; Nordbotten et al., 2008; Romanak et al., 2012), before its widespread deployment takes place. Proper site characterization, monitoring, and pressure management should allow minimizing the risk of perceivable induced seismicity in Gt‐scale CO_2_ injection (Celia, 2017; Rutqvist et al., 2016; Vilarrasa et al., 2019). The considered storage formations to date include deep saline aquifers, depleted oil and gas fields, and unmineable coal seams in which CO_2_ stays in supercritical conditions with a relatively high density but lower than the one of the resident brines (Hitchon et al., 1999). Thus, the risk of CO_2_ leakage, although low (Alcalde et al., 2018), may be present for up to millions of years until all CO_2_ becomes dissolved into the resident brine or mineralized (Benson & Cole, 2008).

A few concepts have been proposed to date to reduce the risk of CO_2_ leakage. These concepts consist either in promoting fast mineralization or storing CO_2_ already dissolved in the resident brine. Regarding rapid CO_2_ mineralization, injecting CO_2_ in shallow basaltic rock allows a quick mineralization thanks to the favorable chemical composition of the host rock, although leakage through buoyancy remains a major concern in the absence of low‐permeable caprocks or whenever the caprock integrity is compromised (Gislason & Oelkers, 2014). Another storage rock for mineralization could be peridotite, in which carbonation occurs naturally when exposed to atmospheric CO_2_ (Kelemen & Matter, 2008). Peridotite is rare at shallow depths, and its total capacity for CO_2_ storage is in the order of Gt, provided that the rock is massively hydraulically fractured to reach all the available mineral. Regarding dissolved CO_2_ storage, the leakage risk is mitigated because brine is heavier when it is CO_2_ saturated (Burton & Bryant, 2009; Sigfusson et al., 2015). CO_2_ dissolution can be performed either on surface (Burton & Bryant, 2009) or at the reservoir depth (Pool et al., 2013). To balance the injection and pumping energetic cost, geothermal heat can be recovered and even electricity could be produced if the temperature is high enough (Pool et al., 2013). However, this storage concept has the drawback that CO_2_ injection capacity is limited by CO_2_ solubility into the brine, which is around 4% at 60°C. Such solubility leads to a storage of roughly 0.1 Mt of CO_2_ per year and per doublet for a circulating brine flow rate of 80 L s^−1^, that is, 2.5 Mt yr^−1^ of water being pumped and reinjected. Thus, very large volumes of brine would need to be circulated—a scenario that makes injection of dissolved CO_2_ only feasible for small‐scale decentralized CO_2_ storage. Overall, the alternatives that have been proposed to reduce the risk of CO_2_ leakage entail a limited storage capacity per well with respect to conventional CO_2_ injection in free phase, which diminishes their attractiveness.

To overcome this limitation, we propose an innovative CO_2_ storage concept that reduces the CO_2_ leakage risk, does not require the presence and integrity of a caprock, and maintains a high storage capacity per well. This concept consists in storing CO_2_ in free phase into supercritical reservoirs, that is, reservoirs where water is in supercritical state. Supercritical reservoirs are found in the deeper part of volcanic areas (depth >3 km), where pressure, *p*, and temperature, *T*, of the pore water are likely to exceed its critical point (*p* > 21.8 MPa and *T* > 374°C for pure water). At water's supercritical conditions, an interesting situation occurs: CO_2_ density is higher than the one of water and thus, sinks. Consequently, a low‐permeable caprock is not needed in deep volcanic areas. Injecting CO_2_ into deeper and hotter reservoirs is a new concept that we propose and we deem possible in the light of the recent achievements in deep drilling in volcanic areas demonstrated at the IDDP‐2 project, in which a 4.5‐km‐deep well has been drilled in the Reykjanes volcanic area, Iceland, reaching supercritical water conditions (Friðleifsson et al., 2017).

We examine the potential of storing CO_2_ in deep volcanic areas where resident water is in supercritical state. First, we analyze the plausible injection conditions at the wellhead that permit injecting CO_2_ with a reasonable compression cost. Next, we explore the CO_2_ sinking potential and quantify the CO_2_ plume shape and injectivity. Finally, we estimate the injection rates that could be achieved and discuss the worldwide CO_2_ storage potential in deep volcanic areas.

## 2 Materials and Methods

### 2.1 Water and CO_2_ Equation of State

The equation of state (EOS) of water and CO_2_ is computed via the C++ library CoolProp (Bell et al., 2014), available at CoolProp (http://www.coolprop.org/). CoolProp employs the Span and Wagner (1996) EOS of CO_2_, which is valid up to 800‐MPa pressure and 1100‐K temperature, and the Scalabrin et al. (2006) viscosity model. The EOS of water is valid up to 1 GPa of pressure and 2000‐K temperature and is taken after Wagner and Pruß (2002), which is based on the IAPWS Formulation 1995. The viscosity of water is taken after Huber et al. (2009).

### 2.2 Temperature, Pressure, and Density Profiles Along the Wellbore

We have implemented an explicit scheme to compute the fluid properties variation with depth along the wellbore. During CO_2_ injection, the cold fluid quenches the well in a relatively short time (days to months), so that at equilibrium a colder annulus forms around the well, hindering heat transfer from the surrounding rock, and the injection process becomes adiabatic (Pruess, 2006). The enthalpy is fixed at corresponding wellhead conditions of pressure and temperature *h*(*z*_0_) = *f*(*p*(*z*_0_), *T*(*z*_0_)), and CO_2_ density is evaluated with CoolProp functions along the discretized (*n* = 1,000 intervals) wellbore depth as a function of temperature and pressure *ρ*(*z*_*i*_) = *f*(*p*(*z*_*i*_), *T*(*z*_*i*_)). At each depth increment *i* + 1, the pressure increase is given by *p*(*z*_*i*+1_) = *p*(*z*_*i*_) + *gρ*(*z*_*i*_)(*z*_*i*+1_ − *z*_*i*_), where *g* is gravity acceleration and *T*(*z*_*i*+1_ − *z*_*i*_) is calculated assuming constant enthalpy *h*(*z*_*i*_) = *h*(*z*_0_).

To compute the initial reservoir in situ conditions of the resident water, the weight of the water column to the corresponding depth is calculated assuming thermal equilibrium with the geothermal gradient, hence the only difference with the described procedure is that *T*(*z*_*i*_) is known a priori.

### 2.3 CO_2_ Plume Calculations

We use both analytical and numerical solutions to compute CO_2_ injectivity (ratio between flow rate and wellhead pressure) and the plume geometry (see supporting information [SI] for more details). For the analytical solution, we use the Dentz and Tartakovsky (2009) solution with the correction to incorporate CO_2_ compressibility effects of Vilarrasa et al. (2010). The CO_2_ plume evolution is computed for a specific injection scenario of temperature and pressure that is deemed to be representative of the application. We assume initial pore fluid pressure of 34 MPa and temperature of 500°C and a pressure buildup at the wellhead of 10 MPa in isothermal conditions. The analytical solution is valid for a confined aquifer scenario, which we have assumed to be 500 or 1,000 m thick. The hypothesis of a confined aquifer represents a lower bound case in terms of injection rate: the structural geology features at depth in volcanic areas are quite uncertain and the presence of low‐permeability structures could be represented by faults, chemically altered layers or magmatic intrusions, but could not be present as well.

## 3 Results

### 3.1 Injection Conditions in the Wellbore

**Figure 1:**
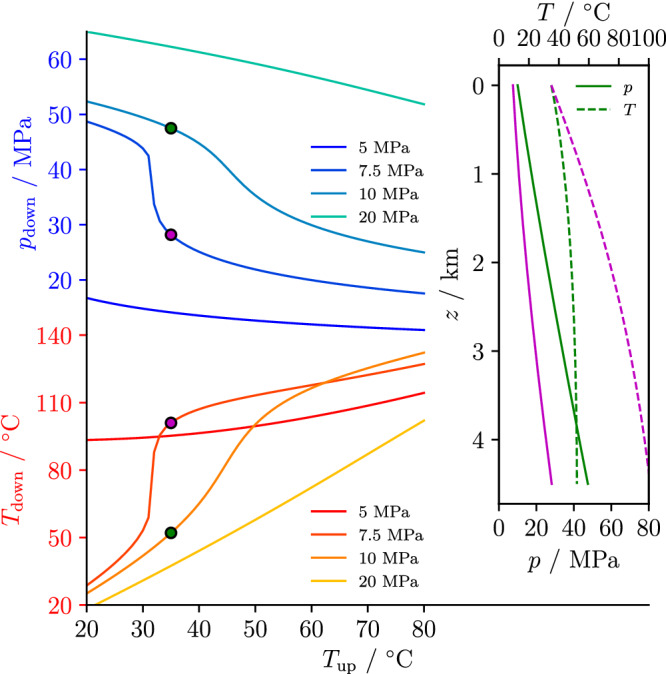
CO_2_ injection conditions at the wellhead and downhole. Each curve shows the pressure, *p*_*down*_, and temperature, *T*_*down*_, conditions at depth of injection (4.5 km) for several wellhead pressures and as a function of wellhead temperature, *T*_*up*_. Injecting CO_2_ at a higher wellhead temperature implies that it reaches the reservoir depth with a lower pressure: In order to ensure injectivity into the rock formation, a minimum downhole pressure threshold should be guaranteed and can therefore be achieved by increasing the wellhead pressure. The sharp transition in the curves corresponding to a wellhead pressure of 7.5 MPa is connected to the phase transition from liquid to supercritical close to the critical point, around which abrupt changes in density take place. The inset displays the evolution of CO_2_ pressure and temperature along the wellbore depth for two different cases, indicated by points in the main figure (color corresponding to two different wellhead conditions). Because of the adiabatic hypothesis, the heating of CO_2_ is a consequence of pressure increase along the wellbore.

CO_2_ downhole pressure and temperature conditions are constrained by limiting reservoir cooling and by ensuring an adequate flow rate through sufficient pressure buildup. Assuming wellbore quenching during continuous injection, the injection temperature and pressure at depth depend on the CO_2_ wellhead temperature and pressure (Figures 1 and S1). According to the EOS of CO_2_, its density is a function of both temperature and pressure and the adiabatic compression generates an increase in CO_2_ temperature with depth (inset in Figure 1). The density profile, in turn, is responsible for the weight of the fluid column, which translates into a pressure increase with depth (Figure S1). At 5 MPa of wellhead pressure, the downhole conditions mildly depend on the wellhead temperature. CO_2_ is strongly heated up by compression along the wellbore because of its high compressibility as it transitions from gas to supercritical fluid (the critical point of CO_2_ is *T* = 31.04°C and *p* = 7.39 MPa) and reaches the reservoir at approximately 100°C and 15–17 MPa, a pressure lower than the one of the reservoirs that prevents CO_2_ flow into the rock. At a wellhead pressure slightly above the critical pressure (see 7.5 MPa in Figure 1), the downhole conditions strongly depend upon the wellhead temperature because of phase transition phenomena. While CO_2_ is in its supercritical phase when injected warmer than its critical temperature, CO_2_ is in liquid phase for cooler injection temperature and reaches the reservoir with higher pressure and lower temperature because of the higher density of the liquid than its gas or supercritical phases. A similar situation occurs when the wellhead pressure equals 10 MPa. At 20 MPa of wellhead pressure, the downhole conditions exhibit small changes between wellhead and downhole temperature because CO_2_ density changes are small at such high pressure.

Downhole overpressure is necessary to ensure that CO_2_ enters into and flows within the reservoir and, if we assume a reservoir pore fluid pressure as in IDDP‐2 of 34 MPa (Friðleifsson et al., 2017), the downhole pressure should not fall below approximately 40 MPa. For example, to achieve such downhole pressure, the wellhead temperature should not exceed 30°C for a wellhead pressure of 7.5 MPa. We can limit reservoir cooling only by injecting at high wellhead pressure and temperature, which implies a high energetic cost.

### 3.2 CO_2_ Sinking Potential

**Figure 2:**
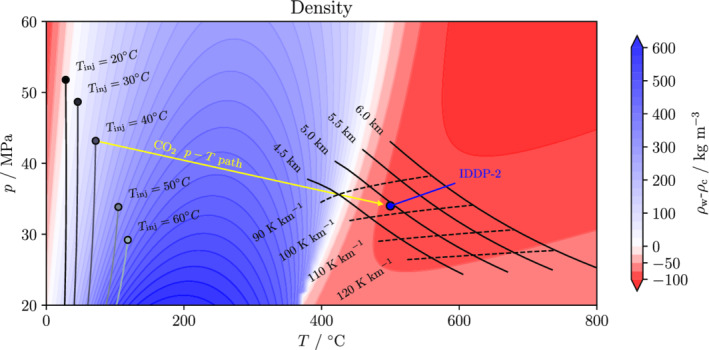
Density difference map between water and CO_2_. The figure shows the density difference between water and CO_2_ as a function of pressure (up to 60 MPa) and temperature (up to 800°C). Positive (in blue) values indicate that CO_2_ has a lower density than water, which leads to CO_2_ buoyancy, and negative (in red) values indicate that CO_2_ has a higher density than water, leading to sinking potential in the reservoir. The downhole conditions of IDDP‐2 are temperature of 500°C and pressure of 34 MPa, which would lead to CO_2_ sinking potential. The dotted black lines indicate the *p*–*T* conditions of a hydrostatic water column for a variety of geothermal gradients and the solid black lines are isodepth for the same case. The trajectories on the left‐hand side indicate CO_2_ injection conditions at the reservoir for several wellhead temperatures and for a wellhead pressure of 10 MPa. The yellow line connects the downhole conditions (buoyant) of a hypothetical injection at IDDP2 with the CO_2_ conditions (sinking) within the reservoir far from the injection well.

Above the critical point of water, both fluids are in supercritical phase and CO_2_ becomes denser than water at increasingly higher pressure as temperature increases (Figure 2). The black solid lines in Figure 2 indicate the pressure and temperature conditions reached by a hydrostatic water column at several depths by taking into account a range of geothermal gradients typical of volcanic areas, indicated with dotted lines. Figure 2 also shows the CO_2_ injection conditions for a wellhead pressure of 10 MPa and several wellhead temperatures along with the estimated in situ conditions of IDDP‐2 of 34 MPa and 500°C (Friðleifsson et al., 2017). For a wellhead pressure of 10 MPa, the maximum wellhead temperature to enable CO_2_ injection is approximately 40°C. At higher wellhead temperature, the CO_2_ density along the wellbore is too small to yield a downhole pressure higher than the one of the reservoirs. Thermal exchange heats up CO_2_ as it flows through the reservoir and CO_2_ temperature and pressure equilibrate to the ones of the reservoir at a given distance from the injection point. The starting and end points of the path (yellow line in Figure 2) in the phase diagram depend upon the reservoir initial conditions and the wellhead injection pressure and temperature. Following our assumptions, the optimum in terms of CO_2_ sinking potential corresponds to gradients between 90 and 120 K km^−1^ and at depths >5 km.

### 3.3 CO_2_ Plume and Injectivity

**Figure 3:**
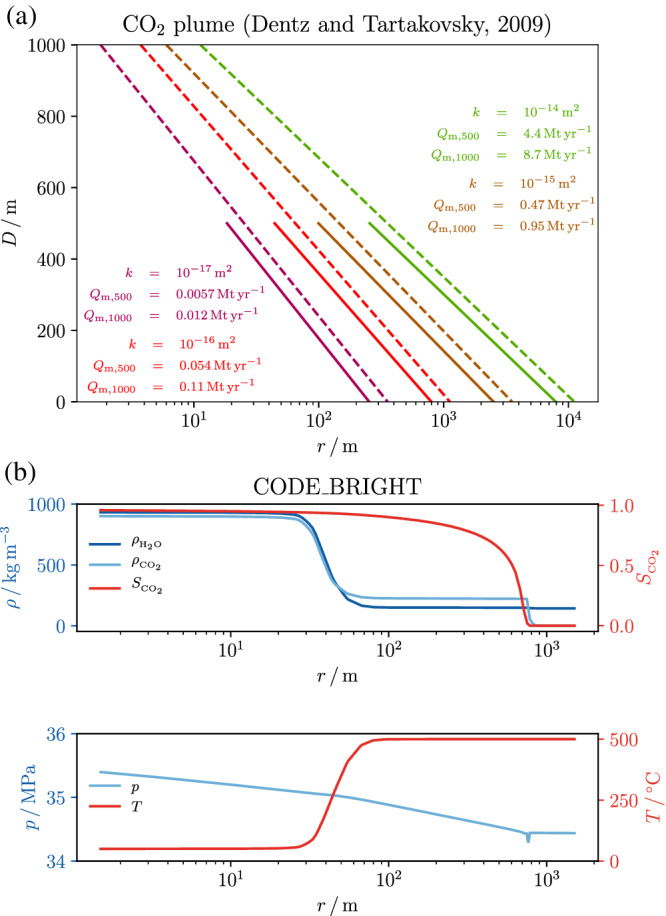
CO_2_ plume. (a) Analytical solutions (Dentz & Tartakovsky, 2009; Vilarrasa et al., 2010) of the CO_2_ plume position for a 10‐year injection into a 500‐m‐thick (solid lines) and 1,000‐m‐thick (dotted lines) reservoirs. We assume a fixed overpressure of 10 MPa at injection, isothermal injection, an initial reservoir temperature and pressure of 500°C and 34 MPa, respectively, and a range of reservoir permeability, *k*, that spans 3 orders of magnitude. The mass flow rate, *Q*_*m*_, is a function of the reservoir permeability and thickness. The analytical solution predicts a sinking profile due to the density difference between water and CO_2_. (b) Simulation results after 10 years of injecting 1.0 Mt yr^−1^ of CO_2_ at 50°C through 500 m of open well centered into a 2,000‐m‐thick reservoir. The extent of the cooled region has a limited size compared to the CO_2_ plume and does not affect its sinking tendency.

The analytical solution of Dentz and Tartakovsky (2009), with the correction of Vilarrasa et al. (2010) applied to consider CO_2_ compressibility effects for accurately computing CO_2_ density within the plume, estimates a downward CO_2_ plume (Figure 3a). We consider a 10‐year injection of CO_2_ over 500‐ and 1,000‐m‐thick reservoirs, assuming a pressure buildup of 10 MPa in a water‐saturated reservoir initially at *p* = 34 MPa and *T* = 500°C. The extension and shape of the plume are a function of the reservoir permeability and thickness, with its maximum located in the lower part of the reservoir. The maximum extension of the downward plume spans over almost 2 orders of magnitude for a range of permeability of 3 orders of magnitude, ranging from approximately 2.5 × 10^2^ m for the least permeable case to approximately 1.0 × 10^4^ m for the most permeable one. The achievable mass flow rate is also proportional to the reservoir permeability and thickness and ranges from 0.0057 to 4.4 Mt yr^−1^ for a 500‐m‐thick reservoir and from 0.012 to 8.7 Mt yr^−1^ for a 1,000‐m‐thick reservoir.

**Figure 4:**
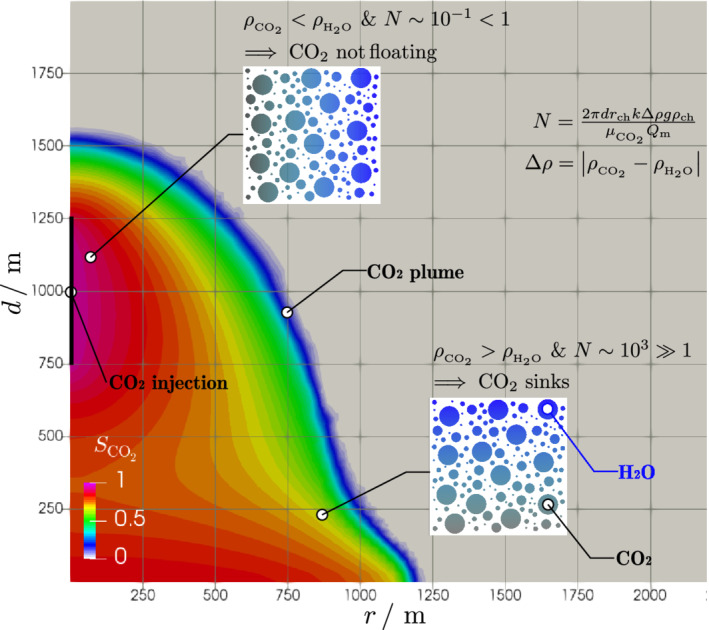
CO_2_ sinking mechanism. The numerically computed sinking profile of CO_2_, represented as the area with CO_2_ saturation *S*_*c*_ > 1, is a consequence of the interplay between gravity and viscous forces as represented by the values of the gravity number *N*. Cold CO_2_ injection does not increase CO_2_ buoyant potential because thermal equilibrium is reached within a small region from the wellbore where viscous forces dominate over gravity forces. At the far field, CO_2_ is in thermal equilibrium with the reservoir, becoming denser than water, and since gravity forces are greater than viscous ones, CO_2_ has the tendency to sink.

The gravity number *N* (Equation S5), which is the ratio between gravity to viscous forces, is computed for the near field (*T* = 50°C and *p* = 44 MPa), that is, close to the injection point, and for the far field (*T* = 500°C and *p* = 34 MPa), that is, the initial reservoir conditions. At the near field, water is liquid with *ρ*_*w*_ = 1,006.3 kg m^−3^ and CO_2_ is supercritical with *ρ*_*c*_ = 940.2 kg m^−3^, which yields a |Δ*ρ*| = 66.2 kg m^−3^ that favors CO_2_ buoyancy. At the far field, both fluids are supercritical, with *ρ*_*w*_ = 138.1 kg m^−3^ and *ρ*_*c*_ = 219.2 kg m^−3^, which yields a |Δ*ρ*| = 81.0 kg m^−3^ that favors CO_2_ sinking. For a 500‐m‐thick reservoir, the gravity number is for the near field and for the far field and for a 1,000‐m‐thick reservoir for the near field and for the far‐field conditions. According to the gravity number values, at the near wellbore range, viscous forces dominate or are in the range of gravity forces and far enough from the injection point, buoyant forces become predominant. Although the near‐field conditions would favor CO_2_ buoyancy, viscous forces are in the same range of buoyant ones, and thus, CO_2_ buoyancy does not take place or is limited in very thick reservoirs. Far from the injection well, buoyant forces dominate over viscous forces, and since CO_2_ has a higher density than water, CO_2_ tends to sink (Figure 4). Finite element analyses of CO_2_ injection further confirm that an uprising CO_2_ plume does not develop near the injection well and that CO_2_ sinks once it reaches thermal equilibrium with the rock (Figures 3b and 4). The cooled region concentrates around the injection well (Figure 3b) and even though CO_2_ is lighter than water within this cold region, no upward flow occurs due to buoyancy. Thus, CO_2_ sinks, leading to a safe storage despite cooling around the injection well.

## 4 Discussion

### 4.1 Challenges

The coupling between the wellbore and the reservoir is important in storage formations with high temperature, like deep volcanic areas. The conflicting objectives of limiting cooling to minimize the risk of inducing seismicity in the long term (Parisio, Vinciguerra, et al., 2019) and of minimizing compression costs by lowering wellhead pressure can only be resolved with accurate optimization procedures. Since CO_2_ density decreases with temperature, the lower the injection temperature, the higher the downhole injection pressure (Figure 2). Thus, a trade‐off arises between the injection pressure and temperature at the wellhead. The optimum injection conditions are site specific and should be computed according to the characteristics of each site. The pressure and temperature injection conditions at the wellhead are coupled to the injectivity of the reservoir and thus to the required pressure buildup at the downhole to inject a given mass flow rate. Given the highly nonlinearity of flow along a wellbore (Lu & Connell, 2014), the wellhead injection conditions will be determined by the injection mass flow rate and the reservoir transmissivity.

Injecting relatively cold CO_2_ (*T* = 20°C) reduces the compression costs because of its higher density (Figure 2). The most energetically efficient option is to inject CO_2_ in liquid state, that is, *T* < 31.04°C (Vilarrasa et al., 2013), a solution that bears the consequence of cooling down the rock in the vicinity of the injection well. Cooling‐induced thermal stress is inversely proportional to the injection temperature and is likely to enhance injectivity (Yoshioka et al., 2019) but also microseismicity by approaching failure conditions: Operators may therefore prefer to inject CO_2_ at a relatively high temperature (40 ÷ 60°C). Heating CO_2_ entails large energetic costs (Goodarzi et al., 2015), which in volcanic areas could be minimized by extracting heat from existing geothermal wells. Injecting hot also increases compression cost because the higher the injection temperature, the higher the required wellhead injection pressure. The energy spent to compress the CO_2_ should have a renewable source to comply with the objective of reducing CO_2_ emissions. Unlike solar or wind resources, which provide time‐fluctuating power output, geothermal energy best fits the purpose of providing a time‐constant heat supply required for continuous CO_2_ injection.

Combining geothermal energy production with geologic carbon storage is of particular interest to utilize the injected CO_2_ and generate a synergy to maximize the cut of CO_2_ emissions in volcanic areas. Exploiting a volcanic area for both geothermal and CO_2_ storage purposes would foster subsurface characterization, reducing uncertainty and identifying the most suitable areas for both geothermal production and geologic carbon storage. CO_2_ could be eventually used as working fluid once the CO_2_ plume has grown enough (Randolph & Saar, 2011).

### 4.2 Managing Risks

The CO_2_ injection rates in deep volcanic areas can be of up to several Mt per year per well (Figure 3a). High injection rates induce pressure buildup and cooling that will in turn affect the geomechanical stability of faults and potentially induce seismic events. Pressure buildup is the main triggering mechanism in the short term and cooling dominates in the long term. The latter may limit the lifetime of injection projects if induced earthquakes become too frequent or of excessively high magnitude (Parisio, Vinciguerra, et al., 2019). The thresholds in frequency and magnitude of induced seismicity are site specific and depend on the local structural and tectonic features. Thresholds to induced seismicity, both in terms of magnitude and frequency, depend on the local conditions and on the consequences produced on the population and infrastructure: The risk might be low in isolated areas but unbearably high in densely populated volcanic areas around the world. In any case, induced seismicity risks should be minimized through subsurface characterization, continuous monitoring, and adequate pressure and temperature management.

The risks of CO_2_ injection in volcanic areas are site specific and should be carefully assessed and evaluated prior to each potential development project. These risks are connected with the intrinsic risks of active volcanism, namely, CO_2_ degassing, hydrothermal explosions, and magmatic eruptions—occurrences that could raise concerns about the feasibility of anthropogenic CO_2_ injection. CO_2_ degassing is naturally present in volcanic areas and usually has its origin at boiling aquifers with superheated steam, which is buoyant (Chiodini et al., 2001). For the injected CO_2_ to leak and eventually reach the surface, it should reverse its sinking tendency and become buoyant. However, our proposal only considers injecting CO_2_ in supercritical reservoirs, which are placed much deeper and at higher temperature and pressure than boiling aquifers. Yet, similarly to what happens in magma chambers, the denser fluid, that is, CO_2_, might migrate laterally outside of the storage formation and encounter different temperature and pressure conditions at which CO_2_ becomes buoyant (Gudmundsson, 2020). Hydrothermal explosions are caused by spinodal decomposition from metastable states leading to fast reequilibration phenomena (Thiery & Mercury, 2009) and the relative risks can be increased by long‐term fluid extraction in geothermal reservoir, where the pressure drop could bring the system closer to metastable states. We argue that injecting CO_2_ will prevent excessive pressure drawdowns and will help maintain a safe distance in the fluid phase space from metastable and dangerous states, where explosive fluid demixing is possible. The risks of magmatic eruptions are strongly linked with the volcanic activity of a specific site. Consequently, volcanic centers with recent eruptive manifestation should be avoided as target areas of deep CO_2_ injection. Avoiding recently active volcanic centers is seldom restrictive in terms of geographical development because supercritical resident brine can be potentially found at drillable depth in several parts of the world where volcanic manifestations are present (Elders et al., 2014). As an example, the Acoculco Caldera Complex has shown no sign of volcanic activity in the form of eruptions and lava flows since approximately 60,000 years ago (Sosa‐Ceballos et al., 2018). Nonetheless, two wells drilled within the Caldera recorded a very high geothermal gradient, with approximately 300°C at 2‐km depth (Calcagno et al., 2018).

The feasibility of this technology is strictly connected to the drilling technology available and to the possibility of reaching pressure and temperature above the critical point of water such that CO_2_ would sink. For geothermal gradients of 30 K km^−1^, the critical point of water would be encountered at around 13‐km depth, which is currently beyond the available drilling technology. In volcanic areas, because of the higher geothermal gradients, the critical point of water is located at the accessible depth of 3 ÷ 4 km (Friðleifsson et al., 2017). Isolating the lower part of the well through proper casing—a great technological challenge per se (Kruszewski & Wittig, 2018)—is also necessary to ensure that CO_2_ is injected at the proper depth.

### 4.3 Perspectives of Technological Development

CO_2_ injectivity is controlled by reservoir permeability, which is highly dependent on temperature. For example, fractured granite has a transition permeability (called elastoplastic), which depends on a threshold mean effective stress, itself a function of temperature (Watanabe, Numakura, et al., 2017). Above the threshold stress, permeability decreases drastically with increasing mean effective stress. In contrast, fractured basalt is stable until high temperature (>500°C) and at 450°C, the observed permeability depends on stress and ranges from 10^−17^ to 10^−16^ m^2^ for a mean effective confining stress of up to 60 MPa (Watanabe, Numakura, et al., 2017). The mean effective stress in the crust strongly depends on the rheology (Meyer et al., 2019; Parisio, Vilarrasa, et al., 2019), and its determination at high depth and temperature remains uncertain. Considering that permeability measurements on laboratory specimens tend to underestimate natural permeability at the geological scale (Neuzil, 1994) and that during drilling of IDDP‐2, all circulation fluid was lost (Friðleifsson et al., 2017), we believe that in situ permeability ranging from 10^−15^ to 10^−14^ m^2^ is possible in the fractured basaltic crust (Hurwitz et al., 2007). Additionally, during injection, the fluid pressure opens up preexisting fractures, while cooling contracts the surrounding rock, generating an additional fracture aperture: Assuming a cubic relationship of transmissivity with fracture aperture (for which fracture permeability is expressed as *k* = *w*^2^/12, where *w* is the fracture aperture), an increase of the fracture aperture of 1 order of magnitude implies an increase of the fracture transmissivity of 3 orders of magnitude. Stimulation techniques have also the potential to achieve higher permeability at depth (Watanabe et al., 2019; Watanabe, Egawa, et al., 2017).

We estimate that suitable injection sites will permit an injection rate ranging from 0.5 to 8 Mt yr^−1^ per well (Figure 3a). Thus, for every 100 wells drilled worldwide in deep volcanic areas for combined geologic carbon storage and geothermal purposes approximately 50 to 800 Mt of CO_2_ would be stored each year without buoyancy‐driven leakage risk. The number of injection wells that will become operative in the next decades is highly uncertain, but to put in perspective, 100 wells would provide a higher amount than what is currently being stored, representing between 1% and 8% of the total worldwide storage target, a nonnegligible contribution to mitigate climate change effects (IPCC, 2018). Our proposal is currently a blue‐sky idea and several challenges need to be addressed in future works, including the exact deployment of the technology, more refined economical and costs/benefit analyses, predrilling geophysical exploration, site monitoring during operation, improvements, and adaptations of drilling technologies.

## 5 Conclusions

We show that storing CO_2_ into reservoirs in which the resident water is in supercritical state will reduce the risk of buoyancy‐driven CO_2_ leakage. Even when CO_2_ is injected much colder than the reservoir temperature, leading to CO_2_ becoming locally buoyant, no buoyant forces arise around the wellbore and a sinking CO_2_ plume develops away from the wellbore. The injectivity per wellbore is relatively high due to supercritical fluid mobility, while overpressure remains low. Continuous injection of CO_2_ over a decade is safe, because cooling only affects a radius in the order of tens of meters from the injection wellbore. Over a longer time span, the expansion of the cooled region might increase local seismicity as faults and fractures respond to thermal induced strains, limiting project lifetime. Our analyses prove that injecting into reservoirs above the critical point of water would constitute a complementary solution to the problem of significantly reducing CO_2_ emissions and would extend the current applicability of geologic carbon storage through the CO_2_ sinking effect that hinders buoyancy‐driven leakage to the surface.

## Conflicts of Interest

There are no conflicts to declare.

## Supporting information

Supporting Information S1

Figure S1

Movie S1

## Data Availability

The calculations are easily reproducible and described in detail in section 2. The FEM code for computation of CO_2_ injection can be downloaded freely online (at https://deca.upc.edu/en/projects/code_bright). The input files for the numerical model can be accessed at the institutional repository Digital.CSIC, which practices FAIR principles (https://digital.csic.es/handle/10261/196740).
