# Supplementary material for: Sinking CO2 in Supercritical Reservoirs
Source: Geophys Res Lett. 2020 Nov 29;47(23):e2020GL090456. doi: 10.1029/2020GL090456 (PMC7780548; doi:10.1029/2020GL090456)
Supplement: Supplementary file 1 — Supporting Information S1 [file GRL-47-e2020GL090456-s001.pdf]

Geophysical Research Letters

Supporting Information for

Sinking CO_2 in supercritical reservoirs

Francesco Parisio[1] and Victor Vilarrasa[2, 3, 4 *]

[1] Chair of Soil Mechanics and Foundation Engineering, Institute of Geotechnics, Technische Universität Bergakademie Freiberg, Germany.
[2] Institute of Environmental Assessment and Water Research (IDAEA), Spanish National Research Council (CSIC), Barcelona, Spain.
[3] Mediterranean Institute for Advanced Studies (IMEDEA), Spanish National Research Council (CSIC), Esporles, Spain.
[4] Associated Unit: Hydrogeology Group UPC-CSIC, Barcelona, Spain.

Contents of this file

Introduction
Supplementary Methods
Supplementary Text
Figures S1
Movie S1

Introduction

We provide in the supporting information the temperature, pressure, density and phase of CO_2 vertical profiles along the injection well during isenthalpic injection for several wellhead pressures (Figure S1). We also include an animation of the CO_2 plume evolution when injecting 1 Mt yr^{-1} of CO_2 during 20 years through 500 m of open well in a 2000 m-thick reservoir (Movie S1).

Supplementary Methods

We numerically solve non-isothermal CO_2 injection in a deep volcanic area using the finite element code CODE_BRIGHT (Olivella et al., 1996), which was extended to simulate non-isothermal CO_2 injection (Vilarrasa et al., 2013). The numerical solution of the fully coupled code CODE_BRIGHT simultaneously solves mass conservation of each phase and energy balance. Mass conservation of both CO_2 and water can be written as (Bear, 1972),

$$\frac{\partial(\varphi S_\alpha \rho_\alpha)}{\partial t} + \nabla \cdot (\rho_\alpha \mathbf{q}_\alpha) = r_\alpha, \qquad \alpha = c, w \tag{S1}$$

where φ [-] is porosity, S_α [-] is saturation of the α-phase, ρ_α [M L^{-3}] is density of the α-phase, t [T] is time, $\mathbf{q}_\alpha$ [L^3 L^{-2} T^{-1}] is the volumetric flux of the α-phase, r_α [M L^{-3} T^{-1}] is the phase change term and α is either CO_2-rich phase, c, or aqueous phase, w. In the numerical simulations, we neglect evaporation of water into CO_2, i.e., $r_w = 0$. The volumetric flux of the α-phase is given by Darcy's law

$$\mathbf{q}_\alpha = -\frac{k k_{r\alpha}}{\mu_\alpha}(\nabla p_\alpha + \rho_\alpha g \nabla z), \qquad \alpha = c, w \tag{S2}$$

where k [L^2] is intrinsic permeability, $k_{r\alpha}$ [-] is α-phase relative permeability, μ_α [M L^{-1} T^{-1}] is α-phase viscosity, p_α [M L^{-1} T^{-2}] is α-phase pressure, g [L T^{-2}] is gravity and z [L] is elevation.

Energy conservation, taking into account the non-negligible compressibility of CO_2, can be expressed as (Nield & Bejan, 2006)

$$\frac{\partial\left((1-\varphi)\rho_s h_s + \varphi\rho_w S_w h_w + \varphi\rho_c S_c h_c\right)}{\partial t} - \varphi S_c \frac{Dp_c}{Dt} + \nabla\cdot\left(-\lambda\nabla T + \rho_w h_w \mathbf{q}_w + \rho_c h_c \mathbf{q}_c\right) = 0 \quad \text{(S3)}$$

where ρ_s [M L^{-3}] is solid density, h_α [L^2 T^{-2}] is enthalpy of α -phase ($\alpha = c, w, s$ and s stands for solid), λ [M L T^{-3} Θ] is thermal conductivity and T [Θ] is temperature. We assume local thermal equilibrium of all phases at every point.

The liquid density is computed as

$$\rho_w = \rho_{w0} \exp\left(\beta\left(p_w - p_{w0}\right) + \alpha_T T\right)\left(1 + \delta\omega_l^{CO_2}\right), \quad \text{(S4)}$$

where the reference water density ρ_{w0} equals 1100 kg m^{-3} for the reference pressure $p_{w0} = 0.1$ MPa, water compressibility is $\beta = 4.5\times10^{-4}$ MPa^{-1}, the volumetric thermal expansion coefficient is $\alpha_T = -4.1\times10^{-3}$ K^{-1}, $\delta = 1 - \frac{\rho_w V_\phi}{M_{CO_2}}$, $V_\phi = 37.51 - 9.585\times10^{-2} T + 8.740\times10^{-4} T^2 - 5.044\times10^{-7} T^3$ (Garcia, 2003), the molecular mass of CO_2 is $M_{CO_2} = 0.044$ kg mol^{-1}, and $\omega_l^{CO_2}$ is the mass fraction of CO_2 into the liquid phase.

We simulate CO_2 injection into a deep volcanic reservoir with initial temperature and pressure at the top of the injection interval of 500 ºC and 34 MPa, respectively. Unlike the analytical solution (Dentz and Tartakowsky, 2009), the injection interval is not immediately bounded by low-permeable layers. Instead, we inject CO_2 distributed through a vertical well that is open along 500 m centered in a 2 km-thick reservoir that could be either fractured basalt or carbonate rock. The reservoir permeability is 10^{-14} m^2, porosity is 2 %, the retention curve has a gas entry pressure of 0.1 MPa and a van Genuchten shape parameter of 0.5 (Van Genuchten, 1980), relative permeability curves follow cubic functions of the α -phase saturation and the thermal conductivity is 2 W m^{-1} K^{-1}. We prescribe a CO_2 mass flow rate of 1.0 Mt yr^{-1}. CO_2 injection temperature is assumed as 50 ºC, a realistic value given the wellbore calculations (Fig. 1). The outer boundary, placed 5 km away from the injection well, maintains hydrostatic pressure.

The ratio of gravity to viscous forces is given by the gravity number N, defined as (Vilarrasa et al., 2010)

$$N = \frac{2\pi r_{ch} dk \Delta\rho g \rho_{ch}}{\mu_c Q_m}, \tag{S5}$$

where for the considered case, $k = 1\times10^{-14}$ m^2, $\Delta\rho$ is the absolute density difference between water and CO_2, $\rho_{ch} = 584.6$ kg m^{-3} is the characteristic density, here assumed as the average CO_2 density between the density in the near field and the far field, r_{ch} is the characteristic length, which is 1 m for the near field and 1000 m for the far field conditions, $d = 500$ m or 1000 m is the aquifer thickness, μ_c is the CO_2 viscosity and Q_m is the mass flow rate of injected CO_2, which equals 4.4 Mt yr^{-1} or 8.7 Mt yr^{-1} for the reservoir thickness of 500 m and 1000 m, respectively. Density and viscosity of water and CO_2 are a function on the pressure and temperature conditions at the near and far field, which are, respectively, $p = 44$ MPa and $T = 50$ °C and $p = 34$ MPa and $T = 500$ °C. The gravity number expresses the relative influence of buoyant forces, taking low values ($N << 1$) when the problem is dominated by the viscous forces and high values ($N >> 1$) when gravity forces dominate.

Supplementary Text

We have compared costs between traditional CCS systems and our proposed solution with or without joint geothermal production. Compression costs are similar for all systems as the transport pipeline delivers CO_2 at the injection site at a pressure of around 10 MPa (Vilarrasa et al., 2013), hence can be neglected. For the comparison, we have assumed a CO_2 market price based on the price of European Union Emissions Trading System as of 11/02/2020, i.e., 22.91 € t^{-1} of CO_2, and a mass injection rate of 2 Mt yr^{-1}. For the traditional CCS system, assuming drilling costs of 2.5 M€ for a 1.5 km-deep well, 1 CO_2 injection well per project and an operational lifetime of 30 years, we estimate a total positive value of 22.9 € t^{-1} of CO_2 stored. For deep volcanic CCS, assuming drilling costs of 30 M€ for each 5 km-deep well, 1 CO_2 injection well and 1 geothermal production well per project and an operational lifetime of 15 years, we estimate a total positive value of 31.9 € t^{-1} of

CO_2 stored, which becomes 21.9 € t^{-1} of CO_2 stored if geothermal production is not considered. Combined with enhanced supercritical geothermal energy (Parisio et al., 2019), geological carbon storage in deep volcanic areas can be a precious contribution to achieve net negative emissions.

References

Bear, J. (ed.) (1972). *Dynamics of fluids in porous media*, Elsevier, New York.

Dentz, M., & Tartakovsky, D. M. (2009). Abrupt-interface solution for carbon dioxide injection into porous media. *Transport in Porous Media*, *79*(1), 15-27.

Garcia, J. E. (2003). Fluid dynamics of carbon dioxide disposal into saline aquifers. PhD thesis, University of California, Berkeley.

Nield, D. A., & Bejan, A. (2006). *Convection in porous media*, Springer, New York.

Olivella, S., Gens, A., Carrera, J., & Alonso, E. E. (1996). Numerical formulation for a simulator (CODE_BRIGHT) for the coupled analysis of saline media. *Engineering Computations*, *13*(7), 87-112.

Parisio, F., Vilarrasa, V., Wang, W., Kolditz, O., & Nagel, T. (2019). The risks of long-term re-injection in supercritical geothermal systems. *Nature Communications*, *10*(1), 4391.

Van Genuchten, M. T. (1980). A closed-form equation for predicting the hydraulic conductivity of unsaturated soils. *Soil Science Society of America Journal*, *44*(5), 892-898.

Vilarrasa, V., Bolster, D., Dentz, M., Olivella, S., & Carrera, J. (2010). Effects of CO_2 compressibility on CO_2 storage in deep saline aquifers. *Transport in Porous Media*, *85*(2), 619-639.

Vilarrasa, V., Silva, O., Carrera, J., & Olivella, S. (2013). Liquid CO_2 injection for geological storage in deep saline aquifers. *International Journal of Greenhouse Gas Control*, *14*, 84-96.

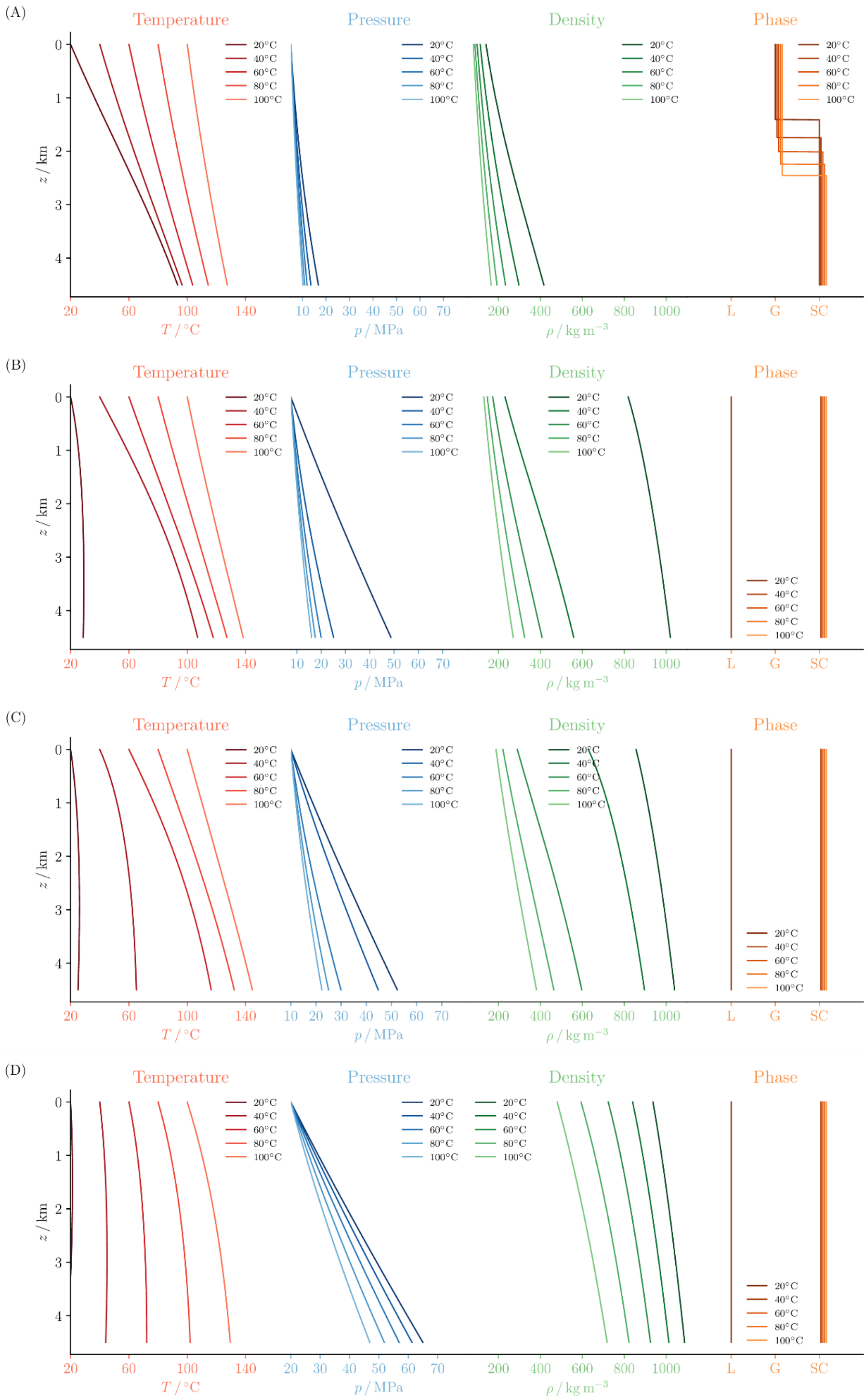

(A)
Temperature
Pressure
Density
Phase
z / km
0 1 2 3 4
20°C 40°C 60°C 80°C 100°C
20 60 100 140
T / °C
10 20 30 40 50 60 70
p / MPa
200 400 600 800 1000
ρ / kg m−3
L G SC
(B)
Temperature
Pressure
Density
Phase
z / km
0 1 2 3 4
20°C 40°C 60°C 80°C 100°C
20 60 100 140
T / °C
10 20 30 40 50 60 70
p / MPa
200 400 600 800 1000
ρ / kg m−3
L G SC
(C)
Temperature
Pressure
Density
Phase
z / km
0 1 2 3 4
20°C 40°C 60°C 80°C 100°C
20 60 100 140
T / °C
10 20 30 40 50 60 70
p / MPa
200 400 600 800 1000
ρ / kg m−3
L G SC
(D)
Temperature
Pressure
Density
Phase
z / km
0 1 2 3 4
20°C 40°C 60°C 80°C 100°C
20 60 100 140
T / °C
20 30 40 50 60 70
p / MPa
200 400 600 800 1000
ρ / kg m−3
L G SC

Fig. S1. Wellbore path of CO_2 injection. Profile of temperature, pressure, density and phase of CO_2 during isenthalpic injection for a wellhead pressure of (a) 5 MPa, (b) 7.5 MPa, (c) 10 MPa and (d) 20 MPa. The phase curves appear slightly shifted to improve visibility in case of superposition, with symbols indicating liquid (L), gas (G) and supercritical (SC) phase.

Movie S1.

Animation of the CO_2 plume evolution when injecting 1 Mt yr^{-1} of CO_2 during 20 years through 500 m of open well in a 2000 m-thick reservoir. The details of the model are described in the Methods, CO_2 plume calculations.
