# Supplementary figures and images for: Sinking CO2 in Supercritical Reservoirs
Source: Geophys Res Lett. 2020 Nov 29;47(23):e2020GL090456. doi: 10.1029/2020GL090456 (PMC7780548; doi:10.1029/2020GL090456)

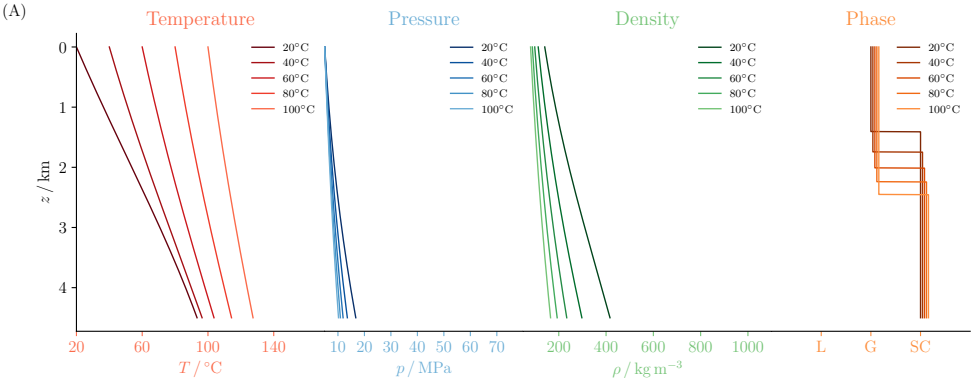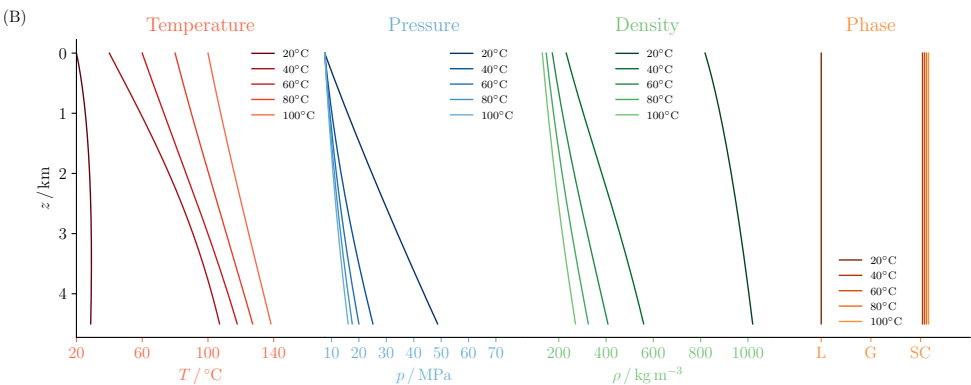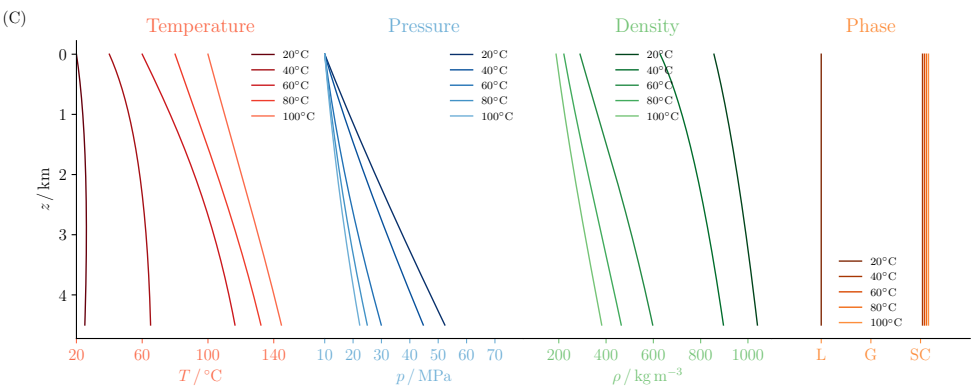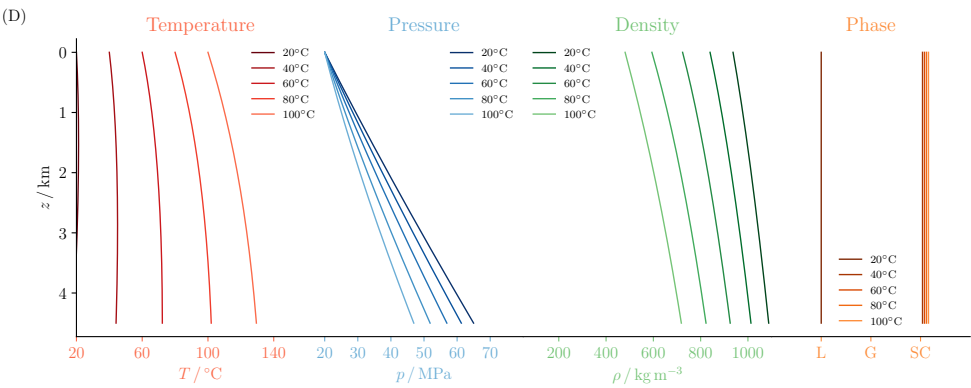

Supplement: Supplementary file 2 — Figure S1 [file GRL-47-e2020GL090456-s002.pdf]
